# Prediction hospital mortality for critical illness lung cancer patients with pneumonia

**DOI:** 10.1186/s12879-025-12484-z

**Published:** 2026-01-14

**Authors:** Caiyun Xu, Jing Li, Zhe Huang, Lan Yao, Huayun Liu, Fuxing Deng, Can Zhu, Qinjuan Jiang

**Affiliations:** 1 Department of Critical Care, Yueyang Central Hospital, No.39 Dongmaoling Road, Yueyang, 414000 China; 2 The First Affiliated Hospital of Xiamen University, No.55 Zhenhai Road, Amoy, 410008 China

**Keywords:** Lung cancer, Pneumonia, In-hospital mortality, Nomogram, Machine learning, Critical care

## Abstract

**Background:**

Pneumonia is a common and severe complication in patients with lung cancer, often resulting in prolonged intensive care stays and increased risk of death. Despite this, no predictive models have been specifically developed for this high-risk population to aid clinical decision-making and early risk identification.

**Methods:**

This study retrospectively analyzed patient data from two large critical care databases: one used for model development and the other for external validation. Adult patients with a diagnosis of lung cancer and pneumonia were included. Clinical features associated with in-hospital death were first screened using single-variable regression, and those with statistical significance were further refined using a variable selection method based on penalized regression. A visual prediction tool was then developed using multivariable regression analysis. Performance was evaluated using standard metrics of discrimination and calibration. Additional machine learning algorithms, including tree-based models, were used to compare performance. Survival analysis was conducted to assess risk grouping capability.

**Results:**

A total of 1046 patients were included in the final analysis. The visual prediction tool incorporated clinical features such as severity scores, mental status assessments, white blood cell count, blood gas indicators, and use of life-support measures. It demonstrated high predictive accuracy (C-index: 0.763) in the external test cohort. The tool outperformed several commonly used machine learning models. Survival curves showed a clear distinction between high-risk and low-risk groups. Calibration and decision analysis confirmed the tool’s clinical usefulness.

**Conclusions:**

This study developed and validated a practical, interpretable prediction model for hospital mortality in patients with lung cancer complicated by pneumonia. The tool enables risk stratification and supports personalized clinical management in intensive care settings.

**Clinical train number:**

Not applicable.

**Supplementary Information:**

The online version contains supplementary material available at 10.1186/s12879-025-12484-z.

## Introduction

Lung cancer is among the most frequent solid tumors encountered in the intensive care units (ICUs), often admitted for respiratory failure or infection [1–3]. Pneumonia – including community-acquired, healthcare-associated, and ventilator-associated types – is a leading cause of ICU admission in lung cancer [4]. The aging population and increased survival rates of patients with cancer have led to a notable increase in the number of patients requiring admission to intensive care units [5]. Indeed, a significant proportion of patients admitted to ICU are afflicted with malignancies, accounting for 20% of cases [6]. Likewise, prospective studies in mixed cancer ICU cohorts find that ICU mortality is 30.6% [3]. A cohort of patients who died with lung cancer had pneumonia in 58.5% of cases [7]. This epidemiologic data emphasizes that pneumonia in lung cancer carries a grave prognosis and contributes substantially to ICU admissions and mortality.

Lung cancer patients carry multiple risk factors for pneumonia: advanced age, smoking-related lung damage or COPD, malnutrition, and treatment-related immunosuppression (chemotherapy, radiotherapy or steroids) increase susceptibility [8–11]. This reflects both tumor effects (obstruction, local inflammation) and the systemic effects (immunosuppression) of lung cancer [12].

Predicting outcomes in lung cancer patients with pneumonia is an important issues. Traditional severity scores, such as Tumor, Node, Metastasis (TNM) [13], Acute Physiology and Chronic Health Evaluation (APACHE) II [14], Simplified Acute Physiology Score (SAPS) II [15], Sequential Organ Failure Assessment (SOFA) have limited accuracy in immunocompromised cancer populations [16]. In ICU lung cancer cohorts, SOFA and SAPS II, have been applied – one study found SAPS II had moderate accuracy (AUC ~ 0.72) for 28-day mortality in lung cancer ICU patients, though performance remains suboptimal. Machine learning (ML) and artificial intelligence (AI) approaches are rapidly advancing [3]. Several studies have developed ML models for pneumonia outcomes. For severe pneumonia in ICU, gradient-boosted trees and neural networks achieved receiver operating characteristics (ROC) > 0.82, outperforming SAPS II [17]. In lung cancer specifically, Huang et al. trained ensemble ML models (random forests, XGBoost, LightGBM) on large ICU databases to predict in-hospital mortality [18]. Their most accomplished collaborative effort yielded optimal area under the curve (AUC) metrics and identified pivotal predictors. The SOFA score, serum albumin, OASIS score, anion gap, and bilirubin were identified as the most salient features. These ML have the capacity to integrate a multitude of variables, including vital signs, laboratory results, and therapeutic interventions, thereby enabling the stratification of risk assessment. The utilization of interpretability methods serves to underscore the significance of features. These AI models show promise for personalized risk prediction, though prospective validation is needed.

To date, very few studies focus exclusively on lung cancer patients with pneumonia in ICU settings. No dedicated predictive model has been developed specifically for pneumonia in patients with lung cancer. In this study, we employed data from two substantial, multi-center ICU databases to construct an interpretable and clinically applicable prognostic model. A Cox regression-based nomogram was developed to predict in-hospital mortality, and its performance was systematically compared with that of several mainstream machine learning algorithms. Furthermore, the employment of model interpretability techniques facilitated the identification and visualization of the most influential clinical factors contributing to mortality risk.

## Methods

### Data source

The Medical Information Mart for Intensive Care IV (MIMIC-IV v3.0) is a large, freely accessible critical care database developed by the MIT Laboratory for Computational Physiology in collaboration with the Beth Israel Deaconess Medical Center [19]. It contains de-identified health-related data associated with over 60,000 ICU admissions between 2008 and 2019. Compared to earlier versions (e.g., MIMIC-III), MIMIC-IV provides a more modular data structure, clearer ontology, improved linkage to hospital administrative systems, and broader temporal coverage. The database includes high-resolution information such as demographics, laboratory tests, vital signs, procedures, medications, and clinical notes, all mapped to standardized coding systems (e.g., ICD-9, 10). Version 3.0 ensures enhanced data consistency and interoperability by separating hospital (core) and ICU-specific data components into distinct modules, facilitating more flexible and accurate longitudinal analyses.

The eICU Collaborative Research Database (eICU-CRD) is a multi-center critical care database developed by Philips Healthcare in partnership with the MIT Laboratory for Computational Physiology [20]. It comprises data from over 200,000 ICU admissions across more than 200 hospitals in the United States during 2014–2015. The dataset includes structured electronic health record data such as patient demographics, diagnoses, interventions, vital signs, laboratory results, and outcomes. Unlike MIMIC, which is based on a single academic center, eICU reflects a geographically and operationally diverse range of institutions, enhancing its generalizability. Data collection is derived from the Philips eICU telehealth system, which enables remote monitoring and real-time decision support, thus offering a unique perspective into critical care delivery in distributed environments.

The individual information of the patients included in the two databases were anonymous, and ethical review and informed consent were waived.

### Cohort selection

Critically ill patients diagnosed with both lung cancer and pneumonia were retrospectively identified from two publicly available critical care databases: MIMIC-IV v3.0 and the eICU-CRD. Patient inclusion was based on International Classification of Diseases (ICD) codes, including both ICD-9 and ICD-10 formats (Supplementary 1) [21–23].

Patients were included if they met the following criteria:

1. Primary diagnosis of lung cancer, determined by relevant ICD codes.
2. Concomitant diagnosis of pneumonia, identified by the presence of qualifying ICD codes within the same hospital encounter.
3. Admission to and ICU.

Exclusion criteria were applied as follows:

1. Age < 18 years at the time of admission.
2. Multiple ICU admissions during the same hospital stay (only the first ICU stay was retained).
3. ICU length of stay < 24 h.

**Fig. 1:**
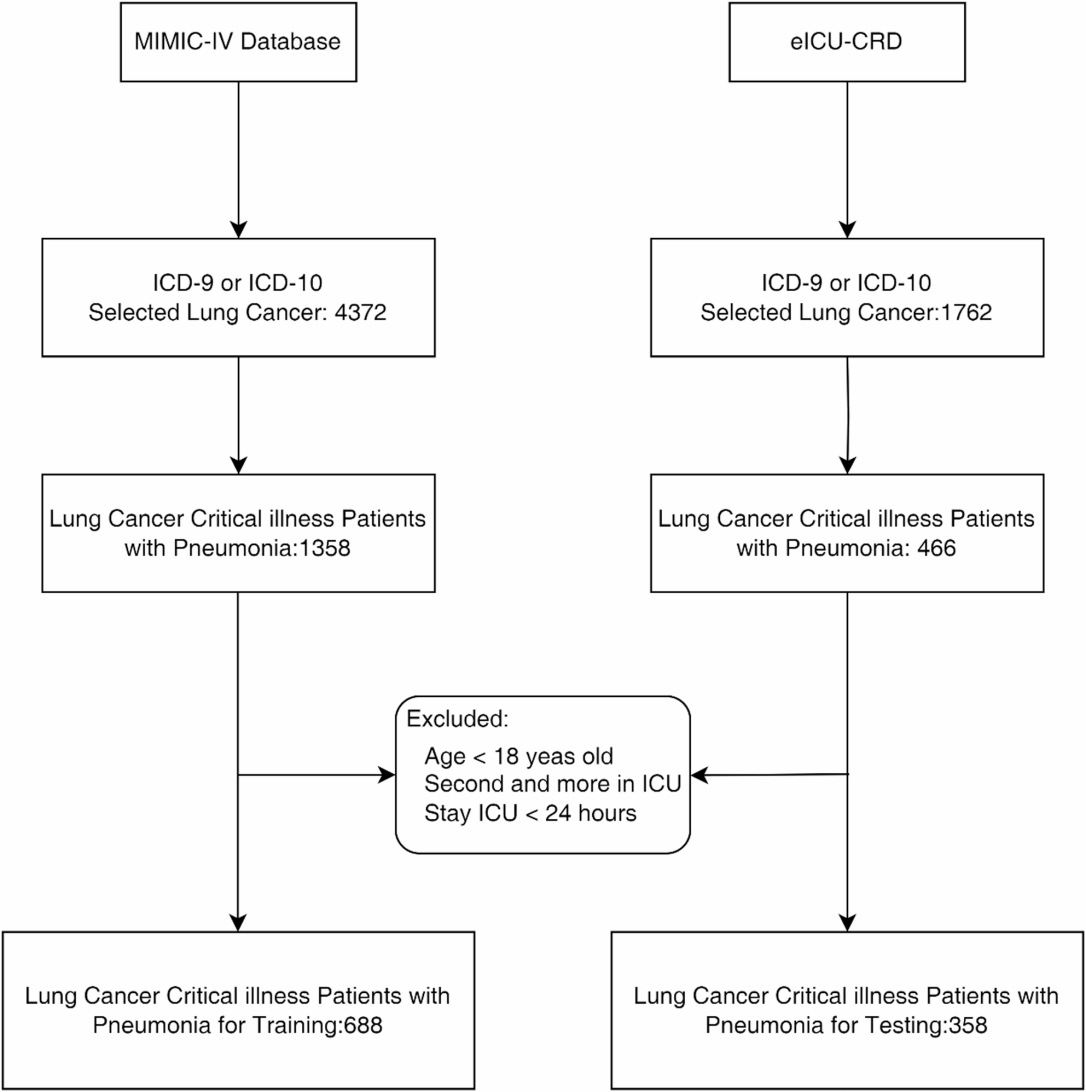
The workflow of patients selection

Following these criteria, a total of 688 patients from MIMIC-IV were included in the training cohort, while 358 patients from eICU-CRD formed the external testing cohort, as illustrated in Fig. 1.

### Date collection and outcomes

Relevant clinical variables were extracted from the MIMIC-IV v3.0 and eICU-CRD databases using PostgreSQL (version 16). Structured query language (SQL) was employed to retrieve patient-level data spanning demographic, and clinical intervention domains during ICU admission. This includes the maximum and minimum values ​​of laboratory and vital signs on the day of admission to the ICU.

The following variables were included for analysis: Demographic and Admission Characteristics: Age, ethnicity, and ICU length of stay (LOS ICU). Severity and Comorbidity Scores: SOFA score, Glasgow Coma Scale (GCS) score, and Charlson Comorbidity Index. Data collection encompassed a broad spectrum of routinely recorded clinical and laboratory variables relevant to the assessment and management of critically ill patients. These were categorized into the following major domains:

Hematologic and Inflammatory Markers: Included complete blood count components such as white blood cell (WBC) count and platelet count, which are essential for evaluating infection, inflammation, and coagulopathy. Renal and Metabolic Function: Parameters such as blood urea nitrogen (BUN), anion gap, lactate, and base excess were used to assess renal perfusion, metabolic acidosis, and systemic hypoperfusion. Arterial Blood Gas Analysis: Routine measurements such as arterial pH, partial pressure of carbon dioxide (pCO₂), and oxygen saturation (SpO₂) were extracted to evaluate respiratory function and acid–base balance. Vital Signs and Hemodynamics: Heart rate, systolic and mean arterial pressure (MAP), and respiratory rate were included as essential indicators of cardiovascular and respiratory status. Comorbidities and Organ Dysfunction: Clinical history was screened for the presence of major comorbidities and complications, including sepsis, paraplegia, metastatic malignancy, and chronic dysfunction of the cardiovascular, renal, central nervous system (CNS), and coagulation systems. Therapeutic Interventions: Information on major ICU treatments such as invasive mechanical ventilation and the use of vasoactive agents (e.g., norepinephrine, dopamine) was recorded to reflect the intensity of care and support needs.

The selected predictors incorporated into the model were chosen not only for their statistical significance but also for their established biological and physiological relevance in critical illness. The SOFA score was included to represent the cumulative burden of multi-organ dysfunction, which encompasses respiratory, renal, hepatic, cardiovascular, coagulation, and neurological components [24]. Higher SOFA scores have consistently been linked with increased mortality, as progressive organ failure reflects the systemic inflammatory and metabolic derangements characteristic of critical care populations. Neurological status, captured by GCS verbal and motor scores, serves as a sensitive indicator of central nervous system compromise [25]. Reduced GCS scores may reflect cerebral hypoperfusion, hypoxia, or sepsis-associated encephalopathy, all of which are independent predictors of poor prognosis in the ICU setting. Metabolic indicators such as anion gap (AG) and base excess (BE) were included to reflect acid–base homeostasis [26]. Elevated AG denotes unmeasured anion accumulation, often due to lactic acidosis or renal dysfunction, while reduced BE indicates buffering depletion—both signaling tissue hypoxia and impaired perfusion in shock or sepsis [27]. Vital sign fluctuations offered additional insights. Higher heart rate (min) and respiratory rate (max) may indicate compensatory physiological responses to systemic stress, while reduced systolic blood pressure (min) and SpO₂ (min) denote circulatory collapse and oxygenation failure. These parameters reflect hemodynamic instability, a hallmark of fatal outcomes in sepsis and multi-organ dysfunction.

Detailed cancer-specific information (such as TNM stage, histologic subtype, molecular markers, prior lines of systemic therapy, and performance status) is incompletely and heterogeneously documented in MIMIC-IV and eICU. Including these variables would have markedly reduced the effective sample size and harmed the external validity of the model. Consequently, the primary objective of data acquisition is to identify the acute, ICU-level physiological derangements that predominantly determine short-term hospital mortality in lung cancer patients with pneumonia. This is achieved indirectly through the accounting of malignancy burden through comorbidity indices (e.g., Charlson Comorbidity Index).

The primary outcome was in-hospital mortality, defined as death occurring at any point during the index hospital admission following ICU admission.

### Construction of in-hospital mortality predictive models

The MIMIC-IV v3.0 cohort was designated as the training set, while the eICU-CRD cohort served as the external validation set to assess model generalizability across independent populations. To develop predictive models for in-hospital mortality, all candidate variables were first assessed for data completeness. Variables with a missing rate exceeding 20% were excluded from further analysis to ensure data integrity. The remaining variables underwent univariate Cox proportional hazards regression, and only those with statistically significant associations (*P* < 0.05) were retained for subsequent modeling steps [28]. Risk stratification was performed using the optimal cutoff value determined by the ROC curve in the training set. Patients with total risk scores above this threshold were categorized as high-risk, while those with scores at or below the threshold were classified as low-risk. This cutoff value was fixed from the training set and applied unchanged to the test set for validation.

To further reduce dimensionality and avoid multicollinearity, Least Absolute Shrinkage and Selection Operator (LASSO) regression was applied [29]. The penalty parameter λ was selected using 10-fold cross-validation, and the final set of predictors corresponded to λ plus one standard error (λ + 1 SE), following the conservative rule to enhance model generalizability. A dynamic nomogram was then constructed based on the selected predictors, allowing for individualized visualization and interpretation of mortality risk in a user-friendly interface [30].

### Machine learning

For comparative purposes, a machine learning framework was also implemented in python 3.8. Predictors from the univariate regression were standardized (z-score normalization) and used as input features for multiple supervised learning algorithms, including Light Gradient Boosting Machine (LightGBM) [31], Extreme Gradient Boosting (XGBoost) [32], Multilayer Perceptron (MLP) [33], and Random Forest (RF) [34]. Model performance was evaluated to identify the optimal approach for mortality risk stratification. These models were developed using the scikit-learn, lightgbm, and xgboost libraries, with hyperparameter tuning performed via grid search and cross-validation on the training cohort (Supplementary 2).

### Statistical analysis and model evaluation

Missing values in predictor variables were handled using multiple imputation by chained equations (MICE) in R. We used the mice package and applied predictive mean matching (PMM) as the imputation method for incomplete variables. Five imputed datasets were generated with 50 iterations (m = 5, maxit = 50; seed = 123), including all candidate predictors in the imputation model. A completed dataset was then derived from the imputed object and used for model development and validation. Baseline characteristics between groups were compared using the independent samples t-test for normally distributed continuous variables or the Mann–Whitney U test for non-normally distributed variables. Categorical variables were compared using the chi-square test or Fisher’s exact test, as appropriate(‘CBCgrps’ packages of R software) [35]. Model performance was assessed on both the training (MIMIC-IV) and external test (eICU-CED) cohorts. Decision Curve Analysis (DCA) was conducted to quantify the net clinical benefit across a range of threshold probabilities for clinical decision-making [36]. For risk stratification, patients were grouped into tertiles (low-/high-risk) based on predicted mortality probabilities. Kaplan–Meier survival curves were generated for each group, and the log-rank test was used to evaluate differences in survival distributions [37]. All statistical tests were two-sided, and a *P*-value < 0.05 was considered statistically significant.

## Results

### Baseline

A total of 1,046 critically ill patients diagnosed with both lung cancer and pneumonia were included in the analysis, comprising 688 patients from the MIMIC-IV database and 358 patients from the eICU Collaborative Research Database. Table 1 summarizes the baseline clinical and physiological characteristics of the two cohorts.

The median age of the study population was 69.5 years (IQR: 61.3–76.7), with no statistically significant difference between the MIMIC-IV and eICU cohorts (*P* = 0.146). The gender distribution was similar across databases, with males comprising 56% of the overall cohort (*P* = 0.319). Regarding ICU severity indicators, the SOFA score was significantly higher in the eICU cohort (median 6 [IQR: 4–8]) compared to the MIMIC-IV cohort (median 4 [IQR: 2–6]) (*P* < 0.001). The GCS score was marginally lower in the eICU patients (median 15 [IQR: 11–15]) than in the MIMIC-IV group (median 15 [IQR: 14–15]) (*P* < 0.001).

Laboratory findings revealed no significant difference in lactate levels between the two databases (median: 1.4 mmol/L, *P* = 0.263). White blood cell (WBC) counts were also comparable between the groups (median: 10.9 × 10⁹/L, *P* = 0.612). However, several markers of organ function and acid–base status, including pH, pO₂, anion gap, and prothrombin time (PT), showed significant inter-cohort differences (*P* < 0.001 for all). The prevalence of sepsis was notably higher in the MIMIC-IV cohort (67%) than in the eICU cohort (43%) (*P <* 0.001). Despite these differences in clinical severity and comorbid burden, in-hospital mortality was not significantly different between the two cohorts—32% in MIMIC-IV versus 29% in eICU (*P* = 0.476). The median LOS ICU was 3.34 days (IQR: 1.91–6.7), with no significant difference observed between the groups (*P* = 0.203).

**Table 1:** Baseline characteristics of critical illness lung cancer patients with pneumonia between MIMIC-IV and eICU-CRD

Variables	Total (*n* = 1046)	MIMIC IV (*n* = 688)	eICU-CRD (*n* = 358)	*P* value
Age, Median (Q1,Q3)	69.53 (61.29, 76.66)	69.55 (61.96, 77)	69 (60, 76)	0.146
Sex, n (%)				0.319
female	459 (44)	310 (45)	149 (42)	
male	587 (56)	378 (55)	209 (58)	
Weight, Median (Q1,Q3)	72 (59.66, 83.30)	71.1 (59, 83.20)	73.9 (60.23, 83.68)	0.291
SOFA, Median (Q1,Q3)	4 (2, 7)	4 (2, 6)	6 (4, 8)	< 0.001
GCS, Median (Q1,Q3)	15 (14, 15)	15 (14, 15)	15 (11, 15)	< 0.001
Charlson comorbidity index, Median (Q1,Q3)	7 (5, 9)	9 (7, 10)	5 (4, 6)	< 0.001
pH, Median (Q1,Q3)	7.37 (7.28, 7.42)	7.35 (7.27, 7.41)	7.39 (7.30, 7.44)	< 0.001
pO_2_,Median (Q1,Q3)	65 (45, 88)	56 (37, 80.25)	73.05 (60.50, 109.07)	< 0.001
pCO_2_,Median (Q1,Q3)	41 (35, 47)	41 (35, 48)	40 (34.20, 46)	0.136
Anion gap, Median (Q1,Q3)	12 (9, 14)	13 (11, 15)	9 (7, 12)	< 0.001
Baseexcess, Median (Q1,Q3)	0 (-3, 3)	0 (-2, 2)	0.4 (-3.10, 3.95)	0.025
Creatinine, Median (Q1,Q3)	0.80 (0.60, 1.20)	0.80(0.60, 1.20)	0.8 (0.60, 1.19)	0.158
Lactate, Median (Q1,Q3)	1.40 (1, 1.90)	1.30 (1, 1.90)	1.40 (1, 1.90)	0.263
PTT, Median (Q1,Q3)	29.60 (26.1, 37)	28.8 (25.80, 32.82)	36.20 (26.90, 61)	< 0.001
INR, Median (Q1,Q3)	1.30 (1.10, 1.50)	1.30 (1.10, 1.40)	1.3 (1.19, 1.78)	< 0.001
PT, Median (Q1,Q3)	14.20 (12.70, 17)	13.90 (12.50, 15.70)	15.3 (13.20, 18.70)	< 0.001
BUN, Median (Q1,Q3)	18 (12, 28)	18 (12, 28)	19 (13, 29)	0.266
WBC, Median (Q1,Q3)	10.9 (7.40, 14.90)	10.9 (7.47, 14.80)	10.9 (6.91, 15.20)	0.612
Heart Rate, Median (Q1,Q3)	78 (67, 90)	78 (66, 90)	78 (68, 91)	0.435
SBP, Median (Q1,Q3)	88 (79, 98)	87 (78, 97)	90.50 (81, 101)	0.001
DBP, Median (Q1,Q3)	47 (40, 54)	47 (39, 53)	49 (42, 55)	0.002
MBP, Median (Q1,Q3)	59 (52, 67)	58 (52, 65)	60 (53, 69)	< 0.001
Respiratory rate, Median (Q1,Q3)	14 (12, 17)	14 (11, 17)	16 (13, 19)	< 0.001
Temperature, Median (Q1,Q3)	36.8 (36.60, 37.09)	36.82 (36.62, 37.08)	36.8 (36.54, 37.1)	0.208
SpO_2_,Median (Q1,Q3)	91 (87, 93)	90 (87, 93)	91 (87, 94)	0.009
Liver disease, n (%)				< 0.001
Non	996 (95)	638 (93)	358 (100)	
Yes	50 (5)	50 (7)	0 (0)	
Diabetes, n (%)				< 0.001
Non	925 (88)	567 (82)	358 (100)	
Yes	121 (12)	121 (18)	0 (0)	
Renal disease, n (%)				< 0.001
Non	911 (87)	553 (80)	358 (100)	
Yes	135 (13)	135 (20)	0 (0)	
Sepsis, n (%)				< 0.001
Non	431 (41)	227 (33)	204 (57)	
Yes	615 (59)	461 (67)	154 (43)	
Ventilation, n (%)				0.247
Non	758 (72)	507 (74)	251 (70)	
Yes	288 (28)	181 (26)	107 (30)	
Vasoactive, n (%)				< 0.001
Non	744 (71)	463 (67)	281 (78)	
Yes	302 (29)	225 (33)	77 (22)	
LOS ICU, Median (Q1,Q3)	3.34 (1.91, 6.70)	3.28 (1.91, 6.04)	3.54 (1.96, 7.62)	0.203
Hospital Expire Flag, n (%)				0.476
Survival	723 (69)	470 (68)	253 (71)	
Dead	323 (31)	218 (32)	105 (29)	

Abbreviation: Abbreviations: Q1, Q3 = First and third quartiles; SOFA = Sequential Organ Failure Assessment; GCS = Glasgow Coma Scale; pH = Potential of Hydrogen; pO₂ = Partial Pressure of Oxygen; pCO₂ = Partial Pressure of Carbon Dioxide; INR = International Normalized Ratio; PTT = Partial Thromboplastin Time; PT = Prothrombin Time; BUN = Blood Urea Nitrogen; WBC = White Blood Cell Count; SBP = Systolic Blood Pressure; DBP = Diastolic Blood Pressure; MBP = Mean Blood Pressure; SpO₂ = Peripheral Oxygen Saturation; LOS ICU = Length of ICU Stay

### Nomogram prediction model

All candidate variables were initially subjected to univariate Cox proportional hazards regression to assess their association with in-hospital mortality. Variables with *P* < 0.05 were considered statistically significant and retained for further modeling. The results of the univariate analysis are summarized in Supplementary Table S1.To reduce multicollinearity and select the most predictive features, LASSO regression was applied to the variables identified in univariate screening. Supplementary Fig. 1 illustrates the LASSO model fitting process.

**Fig. 2:**
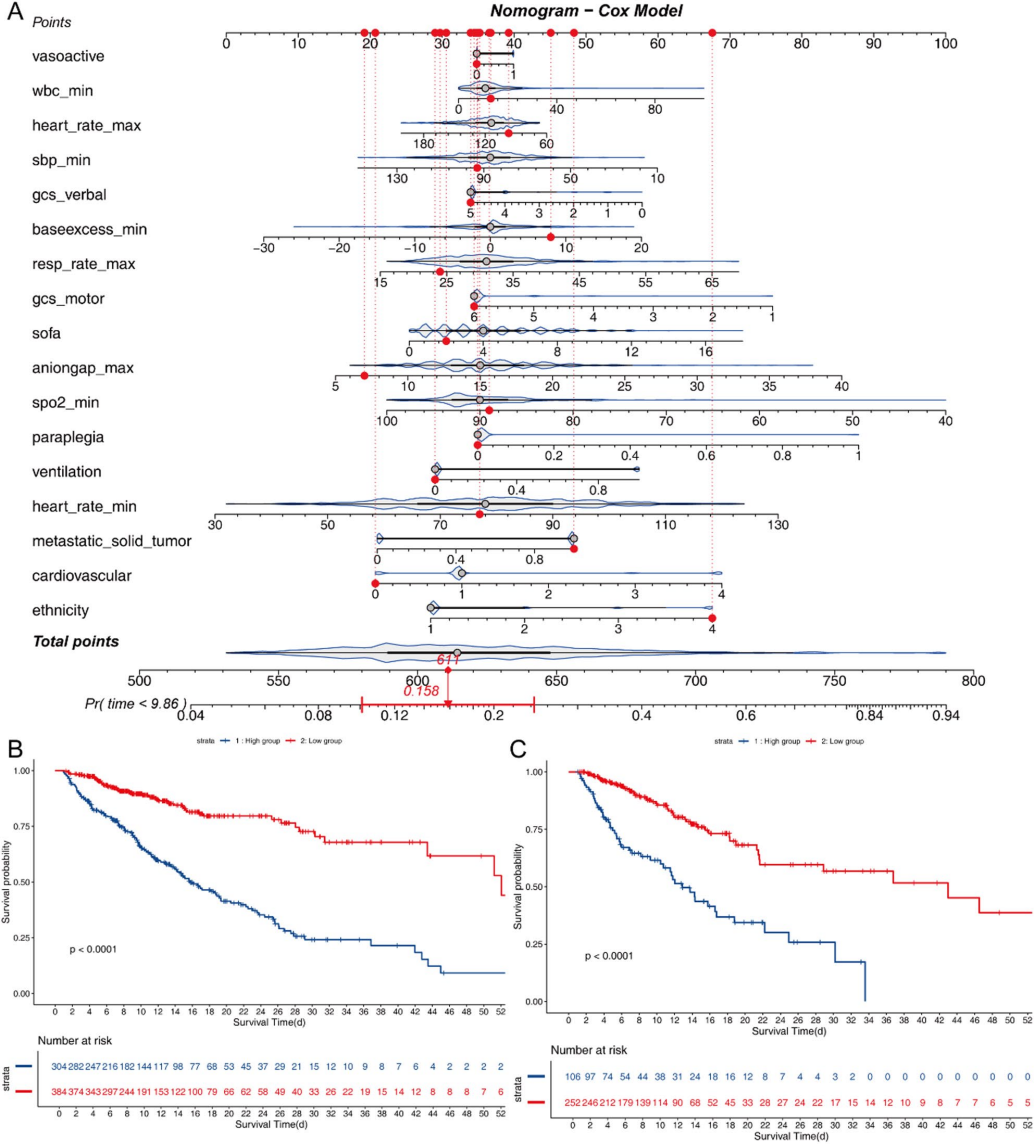
**A**, The nomogram for prediction of hospital mortality among critical illness lung cancer patients with pneumonia; **B**, KM curves according to the risk score of the model for MIMIC IV, classification **C**, KM curves according to the risk score of the model for eICU

A nomogram was developed based on the multivariable Cox regression model incorporating the 17 predictors selected by LASSO regression. Among continuous physiological indicators, higher SOFA scores, base excess (min), anion gap (max), WBC (min), heart rate (min) and respiratory rate (max) were all positively associated with increased in-hospital mortality risk. Conversely, lower scores on the GCS—both verbal (HR: and motor responses—were associated with worse outcomes. Decreased systolic blood pressure (min), heart rate (max) and SpO₂ (min) was also a negative prognostic indicator. In terms of comorbidities and interventions, paraplegia, metastatic solid tumors, and the requirement for mechanical ventilation were associated with higher mortality. Cardiovascular disease, vasoactive medication use, and non-White ethnicity also contributed modestly to increased risk. The resulting nomogram (Fig. 2A) visually represents the relative contribution of each factor, allowing clinicians to calculate individualized survival probabilities based on the cumulative point total assigned to each variable. Higher total scores correspond to increased estimated probabilities of in-hospital death.

Based on the total risk scores derived from the nomogram, patients were stratified into two risk groups: high-risk and low-risk. The ROC-derived cutoff value from training set was 0.430274. Kaplan–Meier survival curves were constructed to compare in-hospital survival between these groups in both the training and validation cohorts. As shown in Fig. 2B, patients in the high-risk group from the training cohort (MIMIC-IV) exhibited significantly lower survival probabilities compared to the low-risk group (*P* < 0.0001, log-rank test), with a hazard ratio (HR) of 3.78 (95% CI: 2.80–5.11). Similarly, in the external validation cohort (eICU-CRD), the survival difference between the two groups remained statistically significant and consistent with the training set, as demonstrated in Fig. 2C (*P* < 0.0001), with an HR of 3.36 (95% CI: 2.27–4.99) relative to the low-risk group.

**Fig. 3:**
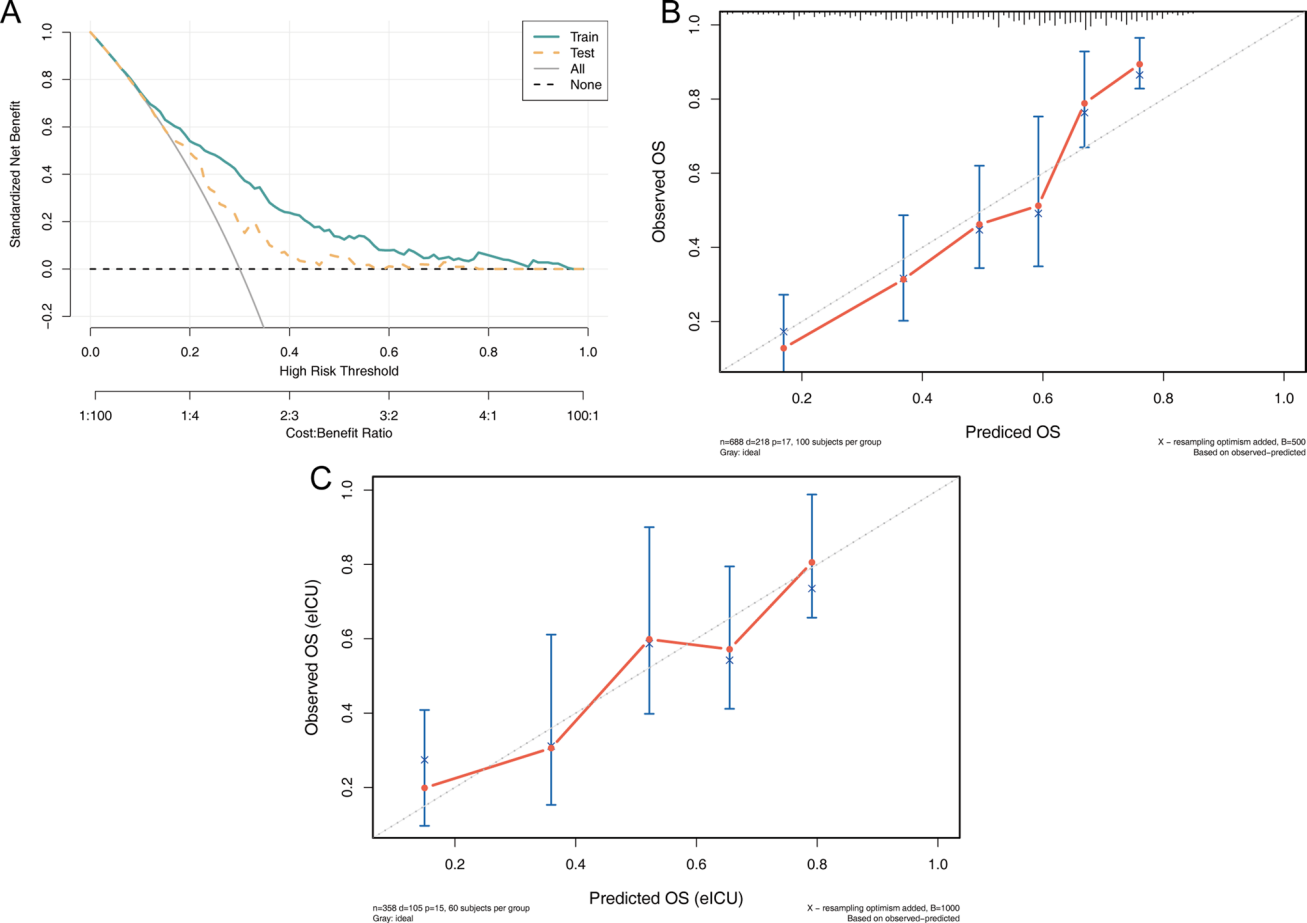
**A**, DCA for Train set and Test set; **B**, Calibration curve of Train set;**C**, Calibration curve of Test set

To assess the calibration of the nomogram, calibration plots were generated comparing the predicted probabilities of in-hospital mortality with the observed outcomes (Fig. 3B&C). The calibration curves in both the training and validation cohorts demonstrated excellent agreement, indicating that the model’s predicted risks were well-aligned with actual patient outcomes. Furthermore, DCA was performed to evaluate the clinical net benefit of the predictive model across a range of threshold probabilities. As illustrated in Fig. 3A, the nomogram showed superior net benefit compared to treat-all or treat-none strategies in both cohorts, confirming its clinical applicability and decision-making value.

### Machine learning

**Fig. 4:**
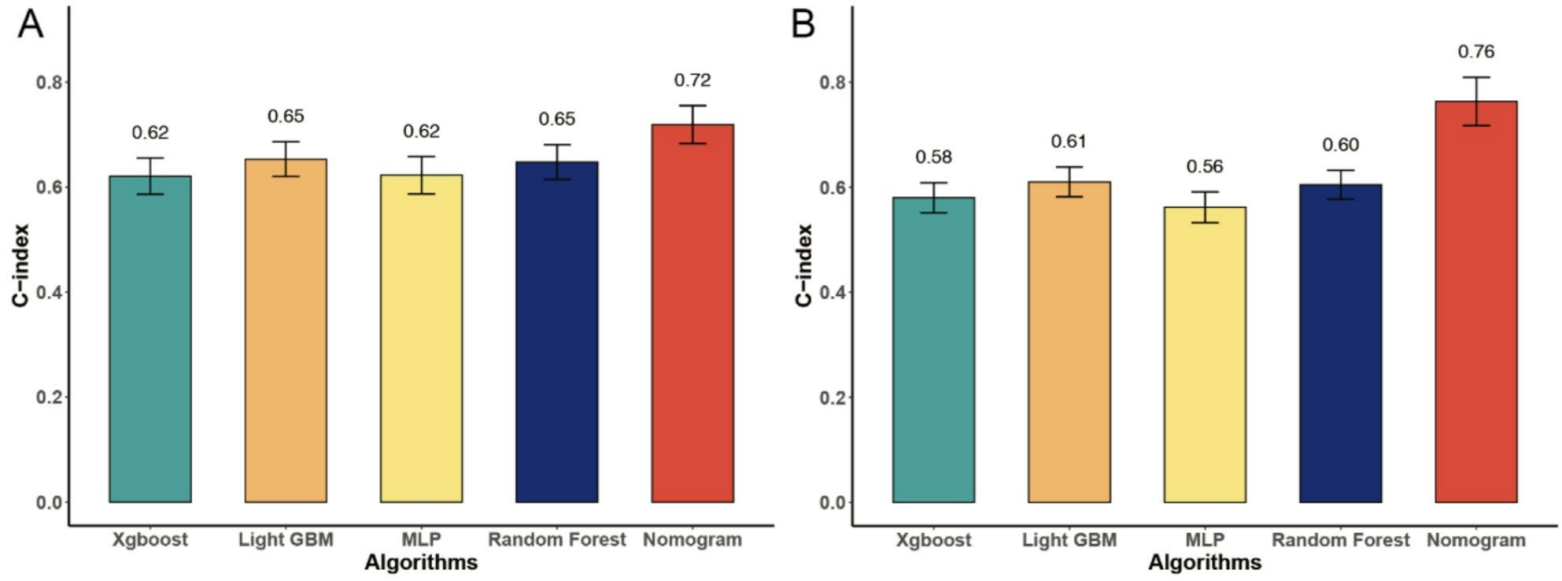
Different ML algorithms of c-indexes. **A**, Train cohort; **B**, Test cohort

To evaluate and compare model discrimination, the C-index was calculated for each predictive algorithm in both the training and test cohorts. In the training cohort (Fig. 4A), the nomogram model demonstrated the highest predictive accuracy, achieving a C-index of 0.719 (95% CI: 0.683–0.755). In comparison, the machine learning models yielded lower C-indices: Light GBM at 0.653, Random Forest at 0.648, Xgboost at 0.621, and MLP at 0.622. As shown in Fig. 4B, the nomogram achieved the highest C-index across all models, with a value of 0.763 (95% CI: 0.717–0.810) in the test cohort, indicating strong discriminatory ability for in-hospital mortality. In contrast, the machine learning algorithms demonstrated lower and relatively similar predictive performance. In the test set, Light GBM yielded a C-index of 0.610 (95% CI: 0.582–0.639), followed by Random Forest at 0.605 (95% CI: 0.577–0.632), Xgboost at 0.580 (95% CI: 0.551–0.608), and MLP at 0.562 (95% CI: 0.533–0.591). These findings underscore the superior accuracy and robustness of the nomogram-based model in predicting in-hospital mortality among critically ill patients with lung cancer and pneumonia, outperforming several widely used machine learning approaches.

## Discussion

In this study, a prognostic model was developed to predict in-hospital mortality among critically ill patients with lung cancer who developed pneumonia, using data derived from the MIMIC-IV and eICU-CRD databases. Following variable screening through univariate Cox regression and LASSO regression, a clinical nomogram was constructed incorporating a range of routinely collected clinical parameters. The nomogram achieved the highest predictive performance among all tested models, with a peak C-index of 0.763 (95%CI: 0.717–0.810) during cross-validation among test cohort, indicating excellent discriminatory ability. Notably, key predictors identified in the final model included the use of vasoactive agents, WBC, heart rate, Systolic blood pressure, GCS verbal response score, and SOFA score. These factors reflect the severity of systemic inflammation, hemodynamic instability, and neurological status. These results support the clinical utility of the proposed nomogram in stratifying mortality risk and guiding early intervention strategies for lung cancer patients complicated by pneumonia in intensive care settings.

Several features underpin the advantages of our model. First, the variable selection process combined univariate Cox regression with LASSO penalization, ensuring parsimony and guarding against overfitting. Second, all predictors are readily available at the bedside or within the first 24 h of ICU admission, facilitating early risk stratification without the need for specialized assays or imaging. Third, the nomogram format translates complex regression coefficients into a visual, user-friendly tool that clinicians can apply in real time to inform discussions about prognosis, triage decisions, and the intensity of monitoring or therapeutic interventions. Finally, by drawing on two geographically and temporally distinct cohorts, our study suggests that the model may retain robustness across different practice settings, although external prospective validation is still warranted.

The inclusion of vasoactive agent use, WBC, heart rate, systolic blood pressure, GCS verbal response score, and SOFA score as key predictors in our nomogram is well grounded in both pathophysiology and intensive care prognostication. Vasoactive support signifies the presence of circulatory failure or septic shock and consistently emerges as a strong mortality indicator in critical illness cohorts, including pneumonia, where its use correlates with increased risk of death [38, 39]. Elevated WBC reflects systemic inflammatory burden and potential sepsis [40]; it frequently appears as a significant predictor in both conventional and machine-learning pneumonia outcome models [41]. Tachycardia and hypotension underscore hemodynamic instability—early warning signs for decompensation and organ failure—and are incorporated into simplified scores like qSOFA, which show good discrimination for mortality [42, 43]. The GCS verbal sub-score provides a sensitive measure of neurological function, with reductions in verbal responsiveness serving as clinically meaningful indicators of sepsis-associated encephalopathy or organ dysfunction [44, 45]. Elevated SOFA scores capture multi-organ failure driven by systemic inflammation and dysregulated immune responses. Severe pneumonia in immunocompromised lung cancer patients triggers a cytokine storm, mediated by IL-6, TNF-α, and NF-κB signaling, which leads to vascular leakage, oxidative stress, and mitochondrial dysfunction, culminating in multiorgan injury [46]. This hyperinflammatory state is often compounded by immune exhaustion secondary to tumor burden and prior chemotherapy, resulting in impaired pathogen clearance and persistent infection [47]. Together, those parameters capture key dimensions of inflammation, hemodynamics, and neurological status, offering a physiologically coherent and clinically actionable basis for mortality stratification in this high-risk patient population.

In the lung–kidney axis, pneumonia-induced acute lung injury can precipitate renal hypoxia and uremic inflammation, creating a self-perpetuating cycle of multi-organ failure [48]. Hemodynamic indicators—including tachycardia, hypotension, and hypoxemia—illustrate microcirculatory collapse and endothelial dysfunction that compromise oxygen delivery to tissues. The resulting hypoxia-reperfusion injury promotes further activation of NF-κB and JAK–STAT pathways, linking systemic inflammation to apoptosis and organ dysfunction [49]. Simultaneously, metabolic indicators such as increased anion gap and decreased base excess signify metabolic acidosis due to lactic acid accumulation, reflecting tissue hypoperfusion and cellular hypometabolism. Dysregulated glucose and mitochondrial metabolism in sepsis-induced lung injury further amplify acidosis through the activation of hypoxia-inducible factor-1α (HIF-1α) and the shift to anaerobic glycolysis [50].

Pneumonia in patients with lung cancer diverges from that seen in other critically ill populations in both pathogenesis and prognostic profile, a distinction that our nomogram further elucidates. In lung cancer, post-obstructive pneumonia—resulting from tumor‐induced airway occlusion—predisposes to localized infection, prolonged symptomatology and cavitation, yet often yields scant microbial isolates, reflecting sterile or mixed inflammatory processes rather than straightforward bacterial invasion [51]. This mechanical obstruction, compounded by radiation‐induced lung injury, smoking‐related immune dysfunction and systemic immunosuppression from anticancer therapies, creates a uniquely hostile pulmonary milieu [52, 53]. Our multivariable Cox‐derived nomogram quantifies these vulnerabilities alongside acute physiologic derangements: rising SOFA scores, extreme base excess and anion gap values, elevated minimum WBC, nadir heart rate, and peak respiratory rate each portend escalating in‑hospital mortality risk, whereas reductions in GCS verbal and motor sub scores, trough systolic blood pressure, peak heart rate and lowest SpO₂ further signal multi‑organ compromise.

Within the restricted context of lung cancer patients admitted to the ICU with pneumonia, short-term hospital mortality is largely driven by the severity of organ dysfunction and acute physiological derangement. Accordingly, our model emphasizes dynamic ICU predictors (e.g., SOFA score, vital signs, gas exchange, metabolic parameters) to estimate immediate risk of death. This ICU-focused risk estimate is intended to complement, rather than replace, cancer-specific prognostic assessments used by oncologists. In future work, extended models that integrate high-quality cancer-specific data (when available) could be developed to provide a more comprehensive, disease-specific prognostic framework.

Despite these strengths, several limitations merit consideration. The retrospective nature of database analyses introduces the potential for unmeasured confounding and selection biases. Certain variables of potential interest—such as detailed oncologic treatment regimens, TNM and KPS scores—were not uniformly recorded or were subject to missingness, which may have limited the model’s comprehensiveness. Furthermore, although cross-validation within the test cohort demonstrated stability, true external validation in independent prospective cohorts is necessary to confirm generalizability. Finally, due to limitations in computational capacity and sample size, the current study did not explore the integration of larger, more complex deep learning frameworks. Future studies incorporating higher-dimensional data and advanced algorithms may further enhance predictive accuracy.

## Conclusion

In conclusion, our nomogram offers a high-performance, interpretable tool for predicting in-hospital mortality among lung cancer patients with severe pneumonia in the ICU. By focusing on readily available clinical parameters, it holds promise for enhancing early risk stratification and guiding tailored interventions in this high-risk population. Further validation and prospective implementation studies will be essential to confirm its impact on clinical decision-making and patient outcomes.

## Supplementary Information

Below is the link to the electronic supplementary material.

Supplementary Material 1

Supplementary Material 2

Supplementary Material 3

Supplementary Material 4

## Data Availability

The datasets generated and/or analyzed during the current study are available in the MIMIC-IV database (https://mimic.physionet.org/iv) and eICU-CRD (https://physionet.org/content/eicu-crd/2.0/).

## Abbreviations

AI: Artificial intelligence
APACHE: Acute Physiology and Chronic Health Evaluation
AUC: Area under the curve
DBP: Diastolic Blood Pressure
DCA: Decision Curve Analysis
eICU-CRD: eICU Collaborative Research Database
GCS: Glasgow Coma Scale
ICD: International Classification of Diseases
INR: International Normalized Ratio
ICU: Intensive care unit
IQR: interquartile range
ML: Machine learning
MBP: Mean Blood Pressure
MLP: Multilayer Perceptron
MIMIC: Medical Information Mart for Intensive Care
LightGBM: Light Gradient Boosting Machine
LOS ICU: Length of ICU Stay
PTT: Partial Thromboplastin Time
PT: Prothrombin Time; BUN: Blood Urea Nitrogen
pH: Potential of Hydrogen
pO₂: Partial Pressure of Oxygen
pCO₂: Partial Pressure of Carbon Dioxide
Q1, Q3: First and third quartiles
RF: Random Forest
SOFA: Sequential Organ Failure Assessment
SBP: Systolic Blood Pressure
SpO₂: Peripheral Oxygen Saturation
TNM: Tumor, Node, Metastasis
WBC: White Blood Cell Count
XGBoost: Extreme Gradient Boosting
