# Supplementary material for: Prediction hospital mortality for critical illness lung cancer patients with pneumonia
Source: BMC Infect Dis. 2026 Jan 14;26:305. doi: 10.1186/s12879-025-12484-z (PMC12888532; doi:10.1186/s12879-025-12484-z)
Supplement: Supplementary file 1 — Supplementary Material 1 [file 12879_2025_12484_MOESM1_ESM.docx]

**Lung Cancer ICD Codes**

- ICD-9: 1620, 1622, 1623, 1624, 1625, 1628, 1629, 20920, 20921, 20923, 20924, 20925, 20926, 20927, 20929, 2312, 2357, 2391
- ICD-10: C33, C3400, C3401, C3402, C3410, C3411, C3412, C342, C3430, C3431, C3432, C3480, C3481, C3482, C3490, C3491, C3492

**Pneumonia ICD Codes**

- ICD-9: 4800, 4801, 4802, 4808, 4809, 481,4820, 4821, 4822, 48230,48231,48232,48239,48240,48241,48242, 48249, 48281, 48282, 48283, 48284,4 8289,4829, 4830, 4838, 4841, 4843, 4846, 4847, 4848, 485,486,4870, 4871,4878,488,4880, 48801, 48802, 48809, 4881, 48811, 48881, 48882, 48889
- ICD-10: J120, J121, J122, J123, J1281, J1282, J1289, J129, J13, J14, J150, J151, J1520, J15211, J15212, J1529,J153, J154, J155, J156, J157, J158, J159, J160, J168, J17,J180, J181, J182, J188, J189, J700, J701, J702,J703, J704, J705, J708, J709, J851, U071
