# Supplementary material for: Prediction hospital mortality for critical illness lung cancer patients with pneumonia
Source: BMC Infect Dis. 2026 Jan 14;26:305. doi: 10.1186/s12879-025-12484-z (PMC12888532; doi:10.1186/s12879-025-12484-z)
Supplement: Supplementary file 2 — Supplementary Material 2 [file 12879_2025_12484_MOESM2_ESM.docx]

Supplementary Table S1. The hospital survival univariate analysis of critical illness lung cancer patients with pneumonia

| **Characteristics** | **Hazard Ratio** | **95%CI** | **P Value** |
| --- | --- | --- | --- |
| Age | 1.01 | 1.00-1.02 | 0.079 |
| Gender | 0.84 | 0.65-1.10 | 0.214 |
| Ethnicity | 1.25 | 1.12-1.40 | <0.001 |
| Weight | 1.00 | 0.99-1.00 | 0.163 |
| LOS ICU | 0.96 | 0.94-0.98 | <0.001 |
| SOFA | 1.14 | 1.09-1.18 | <0.001 |
| GCS | 0.89 | 0.85-0.94 | <0.001 |
| GCS motor | 0.78 | 0.69-0.87 | <0.001 |
| GCS verbal | 0.85 | 0.77-0.95 | 0.003 |
| GCS eyes | 0.76 | 0.64-0.91 | 0.002 |
| Charlson comorbidity index | 1.08 | 1.03-1.13 | 0.002 |
| Lactate min | 1.26 | 1.13-1.39 | <0.001 |
| Lactate max | 1.12 | 1.06-1.18 | <0.001 |
| pH min | 0.11 | 0.04-0.37 | <0.001 |
| pH max | 0.17 | 0.02-1.30 | 0.088 |
| pO2 min | 1.00 | 1.00-1.00 | 0.408 |
| pO2 max | 1.00 | 1.00-1.00 | 0.209 |
| pCO2 min | 1.00 | 0.99-1.01 | 0.995 |
| pCO2 max | 1.01 | 1.00-1.02 | 0.018 |
| Baseexcess min | 0.97 | 0.95-0.99 | 0.008 |
| Baseexcess max | 0.98 | 0.95-1.01 | 0.178 |
| Total CO2 min | 0.99 | 0.97-1.01 | 0.246 |
| Total CO2 max | 1.00 | 0.98-1.02 | 0.916 |
| Coagulation | 1.15 | 1.01-1.31 | 0.041 |
| Cardiovascular | 1.34 | 1.22-1.48 | <0.001 |
| CNS | 1.22 | 1.06-1.40 | 0.005 |
| Renal | 1.12 | 1.01-1.25 | 0.033 |
| Platelets min | 1.00 | 1.00-1.00 | 0.07 |
| Platelets max | 1.00 | 1.00-1.00 | 0.181 |
| WBC min | 1.02 | 1.00-1.03 | 0.025 |
| WBC max | 1.01 | 1.00-1.03 | 0.012 |
| Anion gap min | 1.08 | 1.04-1.13 | <0.001 |
| Anion gap max | 1.06 | 1.03-1.09 | <0.001 |
| BUN min | 1.01 | 1.01-1.02 | <0.001 |
| BUN max | 1.01 | 1.01-1.02 | <0.001 |
| Creatinine min | 1.07 | 0.93-1.24 | 0.323 |
| Creatinine max | 1.05 | 0.94-1.18 | 0.407 |
| INR min | 0.95 | 0.75-1.21 | 0.686 |
| INR max | 1.02 | 0.88-1.17 | 0.837 |
| PT min | 1.00 | 0.98-1.02 | 0.875 |
| PT max | 1.01 | 0.99-1.02 | 0.404 |
| PTT min | 1.01 | 1.00-1.02 | 0.196 |
| PTT max | 1.00 | 1.00-1.01 | 0.111 |
| Heart rate min | 1.01 | 1.00-1.02 | 0.009 |
| Heart rate max | 1.01 | 1.00-1.01 | 0.006 |
| Heart rate mean | 1.01 | 1.01-1.02 | <0.001 |
| SBP min | 0.99 | 0.98-0.99 | <0.001 |
| SBP max | 0.99 | 0.99-1.00 | 0.086 |
| SBP mean | 0.98 | 0.97-0.99 | 0.001 |
| DBP min | 0.99 | 0.98-1.00 | 0.089 |
| DBP max | 1.00 | 0.99-1.00 | 0.215 |
| DBP mean | 0.99 | 0.98-1.00 | 0.123 |
| MBP min | 0.99 | 0.98-0.99 | 0.002 |
| MBP max | 1.00 | 0.99-1.01 | 0.752 |
| MBP mean | 0.98 | 0.97-1.00 | 0.014 |
| Respiritory rate min | 1.01 | 0.97-1.04 | 0.77 |
| Respiritory rate max | 1.02 | 1.01-1.04 | 0.008 |
| Respiritory rate mean | 1.05 | 1.02-1.09 | 0.002 |
| Temperature mean | 0.85 | 0.62-1.16 | 0.311 |
| spO2 min | 0.98 | 0.96-0.99 | 0.003 |
| spO2 max | 0.94 | 0.85-1.05 | 0.272 |
| spO2 mean | 0.93 | 0.88-0.98 | 0.003 |
| Glucose | 1.00 | 1.00-1.00 | 0.82 |
| Urineoutput | 1.00 | 1.00-1.00 | <0.001 |
| Sepsis | 1.62 | 1.19-2.22 | 0.003 |
| Myocardial infarct | 1.37 | 0.96-1.96 | 0.08 |
| Congestive heart failure | 1.18 | 0.87-1.61 | 0.283 |
| Peripheral vascular disease | 1.10 | 0.68-1.76 | 0.701 |
| Cerebrovascular disease | 1.28 | 0.83-1.97 | 0.27 |
| Dementia | 1.26 | 0.47-3.39 | 0.65 |
| Chronic pulmonary disease | 1.00 | 0.77-1.31 | 0.996 |
| Rheumatic disease | 1.51 | 0.80-2.85 | 0.207 |
| Peptic ulcer disease | 0.73 | 0.32-1.65 | 0.454 |
| Mild liver disease | 0.98 | 0.58-1.66 | 0.937 |
| Diabetes without cc | 0.73 | 0.50-1.07 | 0.109 |
| Diabetes with cc | 0.72 | 0.37-1.41 | 0.341 |
| Paraplegia | 2.06 | 1.15-3.68 | 0.015 |
| Severe liver disease | 2.97 | 0.74-12.01 | 0.126 |
| Metastatic solid tumor | 1.49 | 1.12-1.99 | 0.006 |
| AIDS | 0.61 | 0.09-4.33 | 0.619 |
| Colloid vol | 1.00 | 1.00-1.00 | 0.899 |
| Crystalloid vol | 1.00 | 1.00-1.00 | 0.029 |
| Ventilation | 1.72 | 1.29-2.30 | <0.001 |
| Vasoactive | 1.7 | 1.30-2.22 | <0.001 |

Abbreviation：Abbreviations: Q1, Q3 = First and third quartiles; SOFA = Sequential Organ Failure Assessment; GCS = Glasgow Coma Scale; pH = Potential of Hydrogen; pO₂ = Partial Pressure of Oxygen; pCO₂ = Partial Pressure of Carbon Dioxide; INR = International Normalized Ratio; PTT = Partial Thromboplastin Time; PT = Prothrombin Time; BUN = Blood Urea Nitrogen; WBC = White Blood Cell Count; SBP = Systolic Blood Pressure; DBP = Diastolic Blood Pressure; MBP = Mean Blood Pressure; SpO₂ = Peripheral Oxygen Saturation; LOS ICU = Length of ICU Stay.
