# Supplementary material for: Prediction hospital mortality for critical illness lung cancer patients with pneumonia
Source: BMC Infect Dis. 2026 Jan 14;26:305. doi: 10.1186/s12879-025-12484-z (PMC12888532; doi:10.1186/s12879-025-12484-z)
Supplement: Supplementary file 3 — Supplementary Material 3 [file 12879_2025_12484_MOESM3_ESM.docx]

# 1. Random Forest

rf_clf = RandomForestClassifier(

n_estimators=100, # number of trees

criterion="gini",

max_depth=None, # no explicit depth limit

min_samples_split=2,

min_samples_leaf=1,

min_weight_fraction_leaf=0.0

)

# 2. Multilayer Perceptron (MLP)

mlp_clf = MLPClassifier(

hidden_layer_sizes=(100,), # one hidden layer with 100 units

activation="relu",

solver="lbfgs", # as used in our analysis

alpha=0.0001,

batch_size="auto",

learning_rate="constant",

learning_rate_init=0.001,

power_t=0.5,

max_iter=100

)

# 3. XGBoost

xgb_clf = XGBClassifier(

n_estimators=100, # number of boosted trees

objective="reg:squarederror",

max_depth=3,

learning_rate=0.1

)

# 4. LightGBM

lgbm_clf = LGBMClassifier(

num_leaves=31,

max_depth=-1, # no explicit depth limit

learning_rate=0.1,

n_estimators=50, # as specified in the manuscript

subsample_for_bin=200,

min_split_gain=0.0,

reg_alpha=0.0,

reg_lambda=0.0

)
