# Supplementary material for: Prediction hospital mortality for critical illness lung cancer patients with pneumonia
Source: BMC Infect Dis. 2026 Jan 14;26:305. doi: 10.1186/s12879-025-12484-z (PMC12888532; doi:10.1186/s12879-025-12484-z)
Supplement: Supplementary file 4 — Supplementary Material 4 [file 12879_2025_12484_MOESM4_ESM.docx]

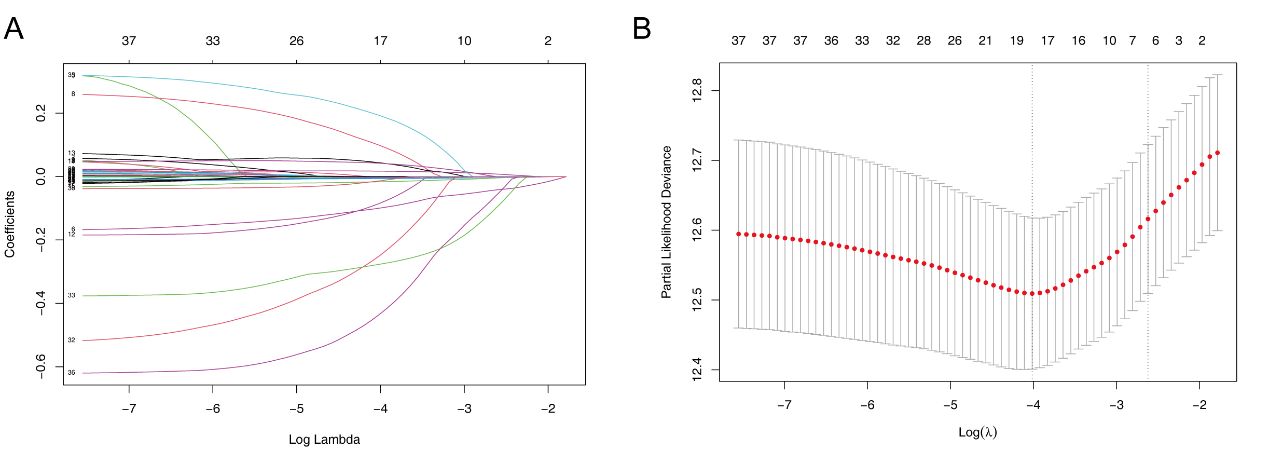
Supplementary Figure 1. Selection of tuning parameter (λ) in the LASSO regression for factors selection. (A) Dotted vertical lines are drawn adopting the minimum rule and the 1 SE of the minimum rule at the suitable values log (λ), where factors are selected. (B) Nine non-zero coefficients are included by LASSO coefficient profiles for clinical factors. LASSO, least absolute shrinkage and selection operator.

Supplementary Figure 1A, coefficient profiles of the candidate variables are plotted against the logarithmic value of the penalty parameter (log λ), showing the shrinkage path of each predictor. As λ increases, more coefficients are compressed toward zero, with only the most robust predictors remaining non-zero. Supplementary Figure 1B displays the results of 10-fold cross-validation used to determine the optimal value of λ. Two dotted vertical lines represent the minimum partial likelihood deviance and the λ within one standard error (1-SE) of the minimum. Based on the 1-SE criterion, a total of 17 variables with non-zero coefficients were selected for final model construction, balancing predictive performance and model parsimony.
